# Population-Based Viral Antibody Profiles of Preschool Children in Burkina Faso

**DOI:** 10.4269/ajtmh.25-0408

**Published:** 2025-11-06

**Authors:** Cindi Chen, Armin Hinterwirth, Mamadou Ouattara, Mamadou Bountogo, Boubacar Coulibaly, Ali Sié, Daisy Yan, YuHeng Liu, Thomas Abraham, Danny Yu, Lina Zhong, Elodie Lebas, Catherine E. Oldenburg, Thomas M. Lietman, Thuy Doan

**Affiliations:** ^1^ Francis I. Proctor Foundation, University of California-San Francisco, San Francisco, California;; ^2^ Centre de Recherche en Santé de Nouna, Burkina Faso;; ^3^ Department of Ophthalmology, University of California-San Francisco, San Francisco, California;; ^4^ Department of Epidemiology and Biostatistics, University of California-San Francisco, San Francisco, California;; ^5^ Institute of Global Health Sciences, University of California-San Francisco, San Francisco, California

## Abstract

Virus-associated infections remain a major burden of childhood morbidity and mortality in sub-Saharan Africa. This exploratory, population-based study used programmable phage immunoprecipitation and sequencing to simultaneously evaluate the antibody response to multiple viruses in dried blood spots from 251 children aged 12 to 59 months who were previously enrolled in the Community Health with Azithromycin Treatment trial conducted in Burkina Faso from 2019 to 2023. Linear mixed effects models, with cluster as the random effect, were used to examine associations between viral antibody response and age, sex, time points (before and after the onset of the severe acute respiratory syndrome coronavirus 2 [SARS-CoV-2] pandemic), and azithromycin mass drug administration (MDA). Sero-reactivity to SARS-CoV-2 was negatively correlated with age in months (β coefficient: −1.43; 95% CI: −2.03 to −0.84; *P_adj_* <0.001), but not to sex (β coefficient: 4.63; 95% CI: −11.90 to 21.17; *P_adj_* = 0.58) or azithromycin MDA (β coefficient: −9.43; 95% CI: −27.56 to 8.71; *P_adj_* = 0.45). Immunoreactivity to the respiratory syncytial virus (RSV) did not appear to be altered after the emergence of SARS-CoV-2 (β coefficient: 39.26; 95% CI: −0.20 to 78.72; *P_adj_* = 0.31). In addition, no detectable differences in the sero-reactivity to poliovirus 1 were observed with azithromycin MDA (β coefficient: 17.86; 95% CI: −25.35 to 61.07; *P_adj_* = 0.82). Although an association was observed between sero-reactivity to SARS-CoV-2 and age, the emergence of SARS-CoV-2 did not appear to alter the antibody response of preschool children in Burkina Faso to RSV or poliovirus vaccine uptake. Longitudinal studies in other at-risk populations in sub-Saharan Africa may improve mechanistic understanding and preventive strategies to decrease childhood morbidity.

## INTRODUCTION

Childhood mortality in some sub-Saharan African countries remains well above the Sustainable Development Goals.^1^ Major attributable causes include infectious diseases.^2^^,^^3^ Although mass drug administration with azithromycin has been shown to decrease childhood mortality in multiple placebo-controlled randomized trials,^4^^–^^7^ not all cause-specific mortality measures are found to be significantly different between the antibiotic-treated communities compared with the placebo-treated communities.^2^^,^^3^ Thus, viral etiology may contribute to the burden of disease but has been inadequately captured. The unbiased characterization of exposure to viral infections may help identify at-risk populations among children to improve prevention strategies.

This study leveraged the biorepository from the Community Health with Azithromycin Treatment (CHAT) trial to evaluate for antibody response to multiple viruses in preschool children in Burkina Faso.^7^ Specifically, dried blood spots (DBS) from children collected in 2019 and 2023 were analyzed using pan-viral phage immunoprecipitation and sequencing (PhIP-Seq) to characterize sero-reactivity to three representative viruses. These viruses were chosen to assess different viral epidemiology: severe acute respiratory syndrome coronavirus 2 (SARS-CoV-2), because it was a new virus that emerged during the study period; respiratory syncytial virus (RSV), because it is a highly prevalent virus associated with substantial mortality in the pediatric population; and poliovirus, which is a surrogate marker for vaccine uptake. This study also evaluated whether azithromycin mass antibiotic treatments influences viral antibody profiles, including responses to poliovirus.

## MATERIALS AND METHODS

### Study overview.

The CHAT trial was a cluster-randomized clinical trial evaluating twice-yearly mass azithromycin distribution (NCT03676764) in the Nouna District of Burkina Faso.^7^ The study was approved by the institutional review boards at the University of California, San Francisco; the Comité National d’Ethique pour la Recherche; and the Comité Technique d’Examen des Demandes d’Autorisation d’Essais Cliniques in Ouagadougou, Burkina Faso. Written informed consent was obtained from the caregiver of each participant.

### Study population.

Children aged 1 to 59 months from a subset of 48 communities were recruited for close monitoring. DBS were collected from a population-based random sample of 15 children per community to assess serologic markers of exposure to pathogens at 0 and 36 months. Baseline samples were collected from September 25, 2019 to November 14, 2019, and 36-months samples were collected from December 29, 2022 to February 21, 2023.

### Phage library and immunoprecipitation of phage-bound antibodies.

The virome peptide library was generated using the UniProtKB database downloaded in January 2024, which contained 381,378 complete protein sequences from 1,963 unique vertebrate animal and human viruses. Open reading frames for each virus were divided into 62–amino acid (aa) peptides, with consecutive peptides sharing a 25–aa overlap. Sequences with 95% identity were collapsed and then converted to DNA sequences. The pan-viral cDNA library was then cloned, packaged, and amplified in T7 phages as previously described.^8^^,^^9^

All samples were de-identified and randomized before processing. Each sample was run in duplicate. Each DBS was resuspended in 200 *µ*L of phosphate-buffered saline (PBS) solution. A 5-*µ*L aliquot of PBS containing antibodies was then incubated with 500 *µ*L of the bacteriophage T7 library (10^10^ plaque-forming units). The participant’s phage-bound antibodies were captured using protein A/G magnetic beads and washed (Supplemental Figure 1). Antibody-bound phages were then eluted and amplified in *Escherichia coli* before a second round of immunoprecipitation. DNA was extracted and sequenced using 150-nucleotide paired-end sequencing on the NovaSeq X (Illumina, San Diego, CA).

### Bioinformatics and quantification.

Sequencing reads were trimmed to remove adapters at the 5′ end and then quality filtered using high throughput sequence analysis (HTStream, v. 1.3.3).^10^ The quality trimmer used a sliding window approach to remove the low-quality ends of reads. Reads with an average quality score <25 over the trim window were removed. When the average quality of the bases within the window was above the threshold, trimming stopped. Only reads with a minimum length of 120 were considered for the alignment step (HTStream parameters: “hts_QWindowTrim –avg-qual-score 25 –window-size 50”, followed by “hts_LengthFilter –min-length 120”). Alignment was performed with Bowtie as part of the phip-flow Nextflow pipeline (Nextflow version 24.10.3.5933, phipflow version 1.14).^11^^,^^12^ Briefly, a Bowtie2 index was created from the peptidome library sequences. Quality-filtered reads were aligned using Bowtie2, allowing a maximum of two mismatches over an alignment length of 100 nucleotides. Tables of counts and normalized counts were generated by phipflow.

The “phippery” library was used to calculate *z*-scores and *P*-values for peptide enrichment using the set of beads-only (mock-IP) control samples as a background model.^12^ Each sample was processed in duplicate. A peptide was called “enriched” if it had a *z*-score >2 and a *P*-value <0.0001 in both duplicates.

Binding affinity to a specific pathogen was determined by aligning to a reference proteome. The pathogen-specific peptides in the phage library were decomposed into 10-mers, with a 2–aa overlap, and each of these k-mers was aligned to the reference proteome using Basic Local Alignment Search Tool for proteins (BLASTp, v. 2.16.0). Normalized read counts (CP100K; peptide-aligned read counts per 100,000 sequencing reads) for enriched peptides were cumulatively summed and mapped to their respective positions on the proteome.

## STATISTICAL ANALYSES

The original sample size calculation for CHAT, used to assess antimicrobial resistance as a primary analysis, targeted 48 communities (24 per arm). This was estimated to provide 80% power to detect a difference in antimicrobial resistance prevalence of 0.082 across arms, assuming a prevalence of 0.05 in the placebo group and an intraclass correlation coefficient of 0.1. Because of regional insecurity, only 41 communities were visited at the 36-month time point. For this subanalysis, 251 samples were randomly chosen from the biorepository, which included all samples collected for the enrolled CHAT communities.

Sero-reactivity for SARS-CoV-2, RSV, and poliovirus 1 (PV1) was defined as the *Log10* of the CP100k across a specified range along the proteome. Linear mixed-effects models were used to assess the relationship between the sero-reactivity of SARS-CoV-2, RSV, and PV1, at the protein-level and proteome-level, and the following predictors: age (months), sex, treatment arm (placebo vs. azithromycin), spike (S) protein sero-reactivity for non-SARS-CoV-2 outcomes, and time of data collection (baseline vs. 36 months). The random effect was the community or cluster to which the child belonged. Statistical significance of each predictor was evaluated using the Wald *t*-test from the linear mixed-effects models. Benjamini-Hochberg false discovery rate (FDR) <0.05 was used to adjust for multiple comparisons, where the family of tests was grouped by outcome.

Linear mixed-effects models were fit using the linear mixed-effects [*lme()*] function from the nonlinear mixed-effects models (*nmle*) package.^13^ A two-sided α of 0.05 was used to determine statistical significance. Data were analyzed using R version 4.3.1 (R Project for Statistical Computing, Vienna, Austria).

## RESULTS

**Figure 1.**
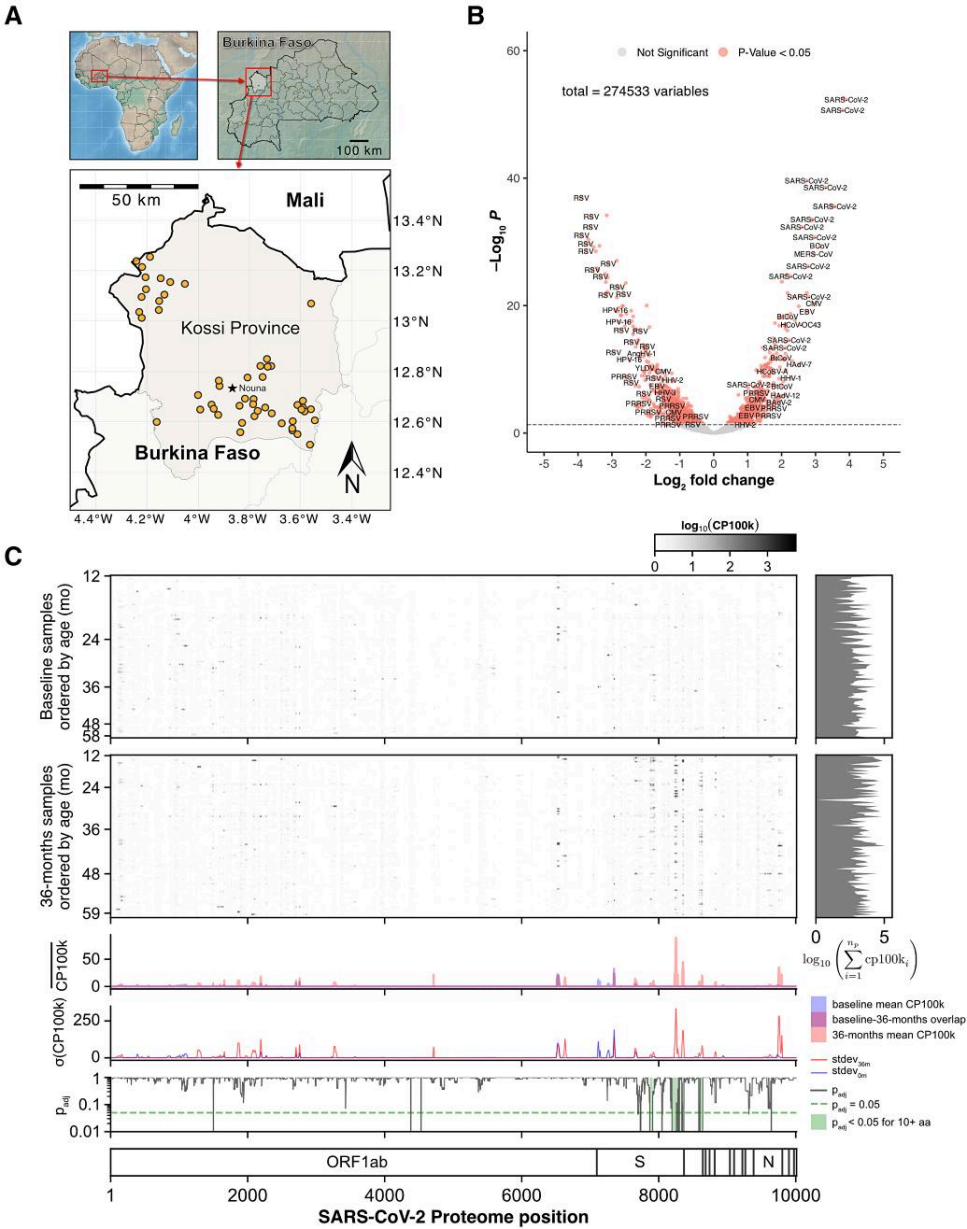
Population sero-reactivity to viruses in Burkina Faso children. (**A**) Geographic distribution of morbidity included in Community Health with Azithromycin Treatment (CHAT) Study. (**B**) Volcano plot of the relative abundance distributions of the viral antibodies of preschool children before and after the severe acute respiratory syndrome coronavirus 2 (SARS-CoV-2) pandemic. The *x* axis shows the log_2_-fold of the relative abundance ratio of antibodies detected from dried blood spots collected from preschool children in 2019 and those collected in 2022–2023. The *y* axis shows the negative log_10_ of adjusted *P*-values. Horizontal dashed line indicates *P* = 0.05. Positive fold change indicates more abundance in dried blood spots collected after the pandemic, and negative fold change indicates more abundance before the pandemic. (**C**) Sero-reactivity across the SARS-CoV-2 proteome in preschool children. Significantly enriched peptides are aligned to the SARS-CoV-2 peptidome. Each row represents the antibody response of a child and is organized in ascending order by age for each time point. The mean peptide enrichments are shown for samples collected at baseline (2019 prepandemic) and at 36 months (2022–2023 postpandemic), with statistically notable differences in 10 amino acid increments highlighted in green. CP100k = peptide-aligned read counts per 100,000 sequencing reads; ORF1ab = open reading frame 1ab.

Antibody responses from 251 children across 52 communities in Burkina Faso were assessed. To minimize the detection of residual maternal antibodies in this study population, we evaluated children aged ≥12 months. Demographic characteristics of those children are summarized in Tables 1 and 2. The median age was 33 months (interquartile range [IQR] = 22–44), and 44% were female. Geographic locations of the sample collection are shown in Figure 1A.

**Table 1:** Demographics of children analyzed at baseline

Demography	Azithromycin	Placebo	Total
No. of clusters	23	18	41
No. of children	69	54	123
No. of children per cluster* Mean (SD)	3 (0)	3 (0)	3 (0)
Sex (%)			
Female	31 (44.9)	24 (44.4)	55 (44.7)
Male	38 (55.1)	30 (55.6)	68 (55.3)
Age, Months			
Mean (SD)	27.16 (11.87)	31.63 (12.64)	29.12 (12.37)
Median (IQR)	23.00 (18.00–36.00)	31.00 (24.00–40.75)	26.00 (18.00–39.00)
Range	12.00–58.00	12.00–58.00	12.00–58.00

IQR = interquartile range; SD = standard deviation.

* Villages were designated as a cluster or split into more than one cluster.

**Table 2:** Demographics of children analyzed at 36 months

Demography	Azithromycin	Placebo	Total
No. of clusters	22	21	43
No. of children	66	62	128
No. of children per cluster* Mean (SD)	3 (0)	2.95 (0.22)	2.98 (0.15)
Sex (%)			
Female	27 (40.9)	29 (46.8)	56 (43.8)
Male	39 (59.1)	33 (53.2)	72 (56.2)
Age, Months			
Mean (SD)	38.61 (12.39)	37.00 (13.46)	37.83 (12.89)
Median (IQR)	38.50 (29.25–49.00)	37.50 (27.25–49.25)	38.00 (28.75–49.25)
Range	14.00–59.00	12.00–59.00	12.00–59.00

IQR = interquartile range; SD = standard deviation.

* Villages were designated as a cluster or split into more than one cluster.

**Figure 2.**
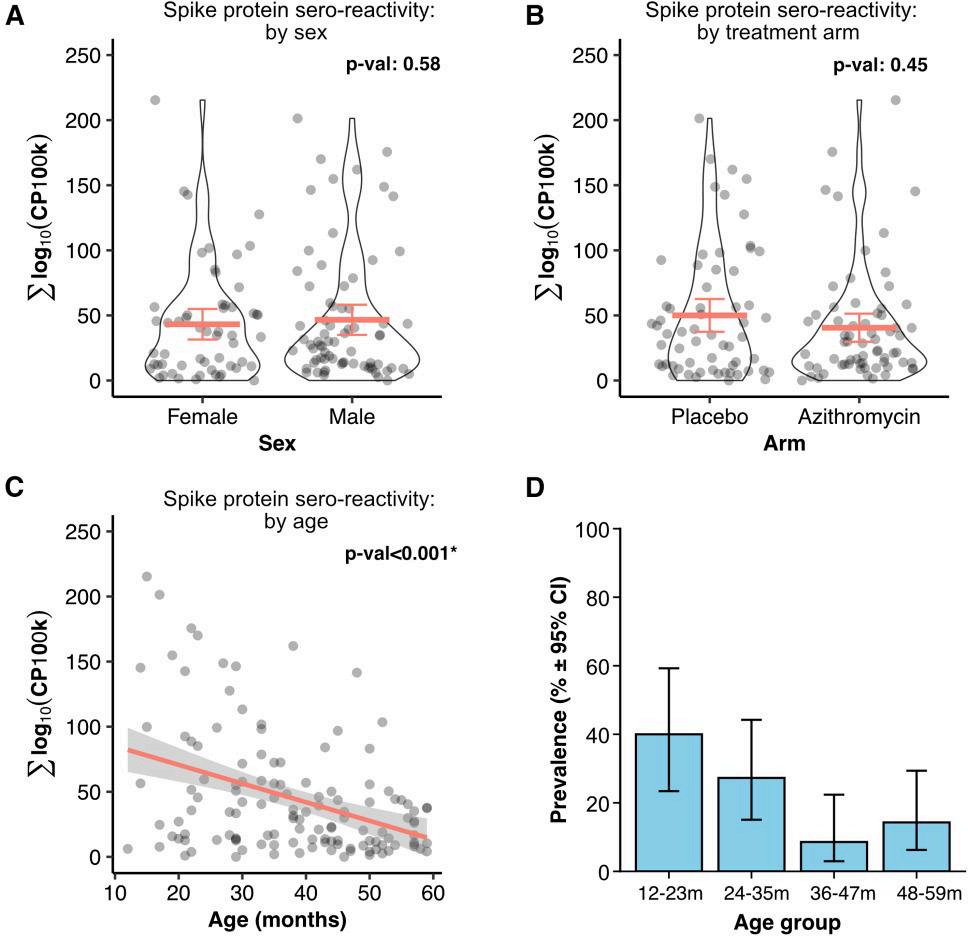
Severe acute respiratory syndrome coronavirus 2 (SARS-CoV-2) sero-reactivity is age dependent. (**A**) Sero-reactivity to the S protein as a function of sex, (**B**) treatment arms, and (**C**) age. Each dot represents the sero-reactivity of a child. Mean and 95% CI are shown for the violin plots. Thick red line shows the regression line, and thin gray lines show 95% of the fit. *P*-values computed from a linear mixed-effects model, with cluster as a random effect. (**D**) Population prevalence of sero-reactivity to SARS-CoV-2 as a function of age. Cumulative sero-reactivity to the S protein was used to determine immunity, with a threshold cutoff set at <5% baseline positivity. 95% CIs were calculated using the Wilson score. CP100k = peptide-aligned read counts per 100,000 sequencing reads.

Differential analysis of the viral antibody response showed that SARS-CoV-2 antibodies were more abundant in the DBS collected in 2022–2023 compared with those collected in 2019 (Figure 1B). The sero-reactivity to the SARS-CoV-2 proteome across all DBS is shown in Figure 1C. Specifically, the sero-reactivity to the S protein accounted for much of the variance detected in DBS collected in 2022–2023. The S protein sero-reactivity was dependent on age by month, with the highest cumulative signal in children aged 12–23 months and the lowest signal in those aged 36–59 months (β coefficient: −1.43; 95% CI: −2.03 to −0.84; *P_adj_* <0.001; Figure 2; Supplemental Table 1). No detectable differences in sero-reactivity were observed in other regions of SARS-CoV-2, including the nucleocapsid protein N (Supplemental Table 2). Furthermore, there were no significant differences in changes in SARS-CoV-2 antibody responses between children from communities receiving azithromycin twice a year for 3 years and those from placebo communities (β coefficient: −9.43; 95% CI: −27.56 to 8.71; *P_adj_* = 0.45; Figure 2; Supplemental Table 1).

**Figure 3.**
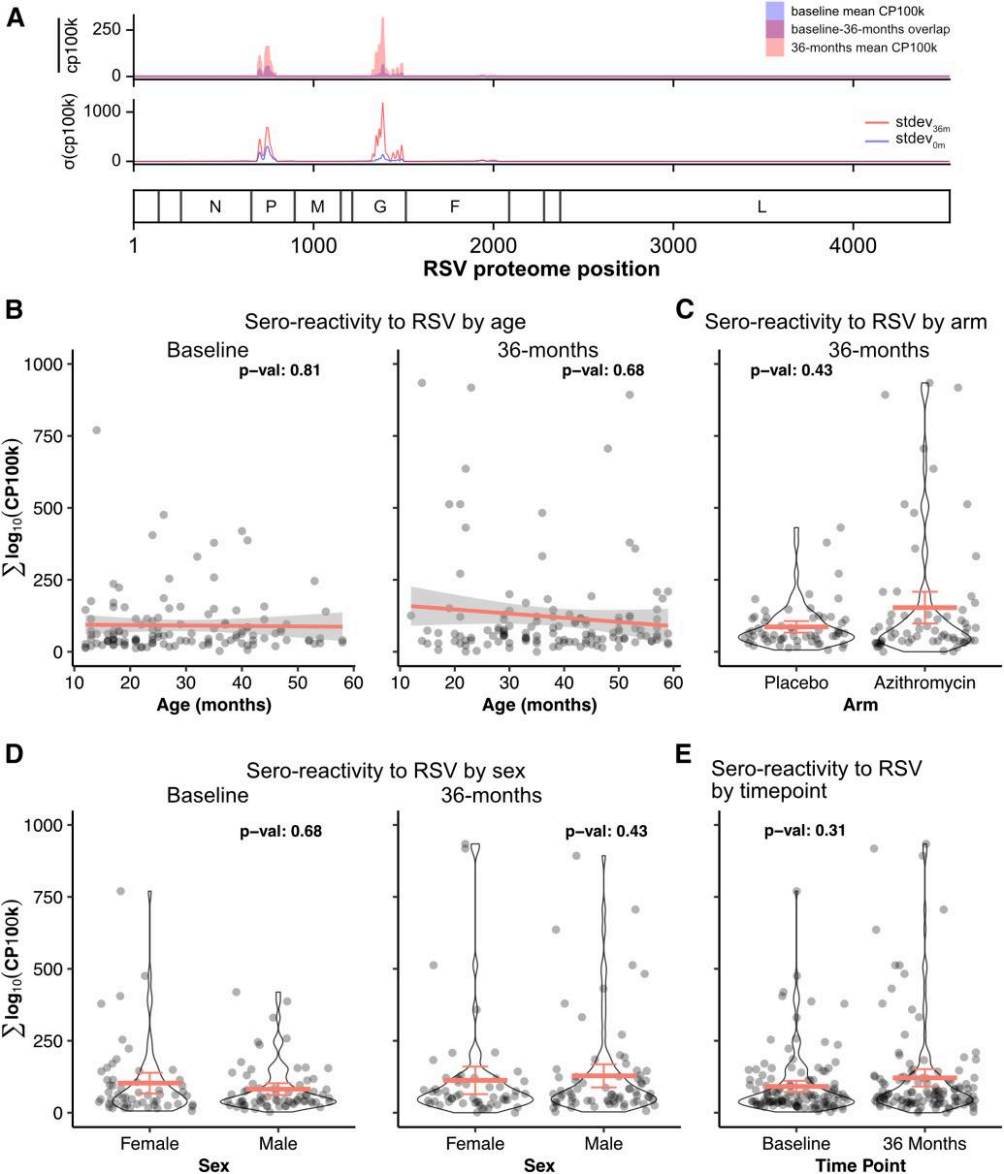
Sero-reactivity to respiratory syncytial virus (RSV). (**A**) Enriched peptides are aligned to the RSV peptidome. The top panel shows the mean peptide enrichments for dried blood spot samples collected at baseline (blue) and at 36 months (pink). The bottom panel shows the standard deviation of the sero-reactivity across the RSV proteome. Sero-reactivity to RSV as a function of age (**B**), treatment arms (**C**), sex (**D**), and time points (**E**). Each dot represents the sero-reactivity of a child. Mean and 95% CI are shown for the violin plots. *P*-values computed from a linear mixed-effects model, with cluster as a random effect. CP100k = peptide-aligned read counts per 100,000 sequencing reads.

We next characterized the sero-reactivity to RSV. Among children aged 12–59 months, antibody response RSV proteome across all samples were mainly driven by sero-reactivity to the phosphoprotein P and glycoprotein G (Figure 3A). The sero-response was not dependent on age or sex (Figure 3; Supplemental Table 3). No significant increase in RSV sero-reactivity was observed at 36 months compared with baseline (β coefficient: 39.26; 95% CI: −0.20 to 78.72; *P_adj_* = 0.31; Supplemental Table 3). Furthermore, children in communities that received six biannual azithromycin treatments did not have measurable changes in RSV sero-reactivity (β coefficient: 54.55 (95% CI: −19.12 to 128.22; *P_adj_* = 0.43; Supplemental Table 3).

**Figure 4.**
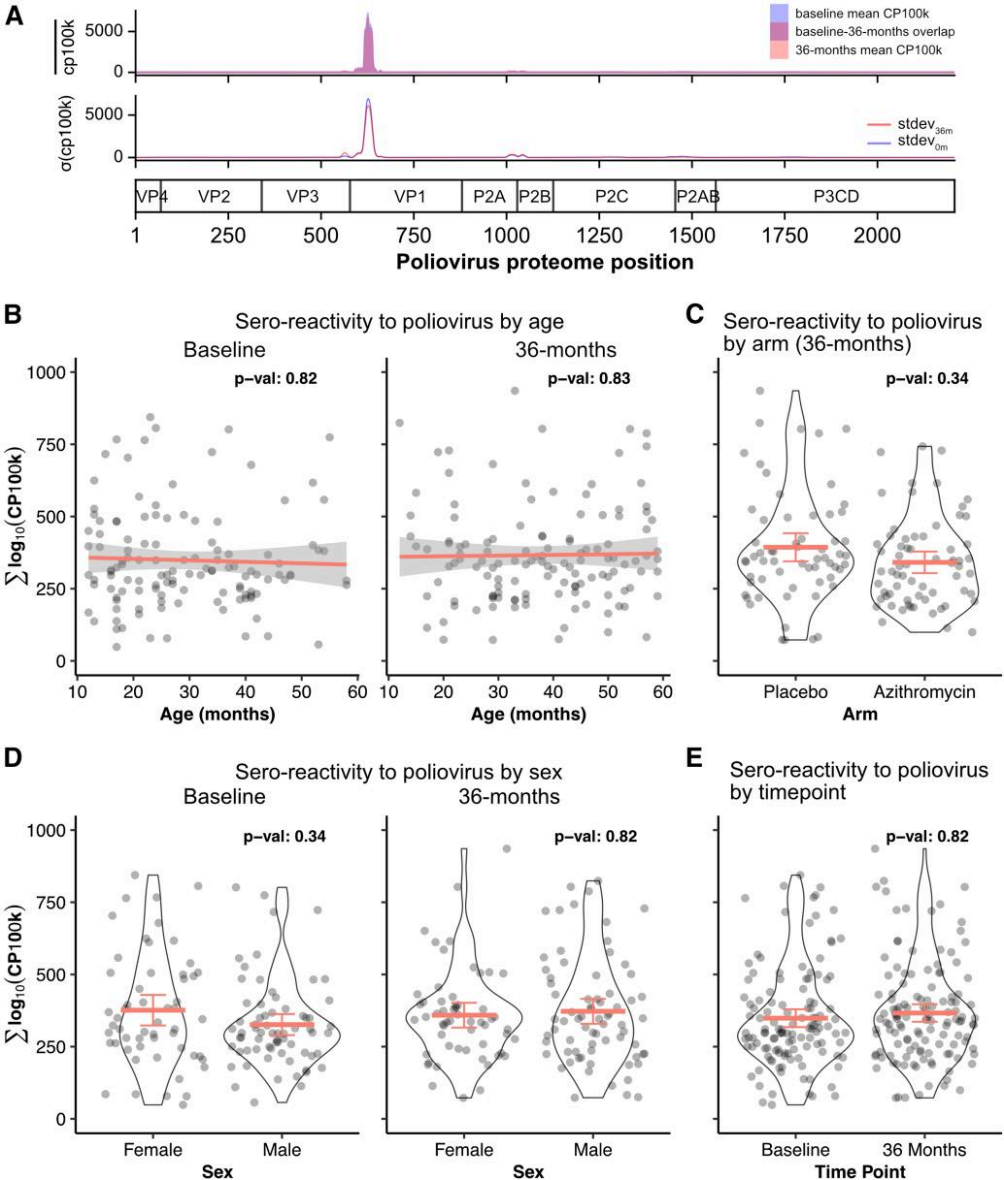
Sero-reactivity to poliovirus 1 (PV1). (**A**) Enriched peptides are aligned to the PV1 peptidome. The top panel shows the mean peptide enrichments for dried blood spot samples collected at baseline (blue) and at 36 months (pink). The bottom panel shows the standard deviation of the sero-reactivity across the PV1 proteome. Sero-reactivity to PV1 as a function of age (**B**), treatment arms (**C**), sex (**D**), and time points (**E**). *P*-values computed from a linear mixed-effects model, with cluster as a random effect. CP100k = peptide-aligned read counts per 100,000 sequencing reads.

In Burkina Faso, children ≤5 years receive free child wellness visits and healthcare. The bivalent oral vaccine (bOPV), containing poliovirus types 1 and 3 (PV1 and PV3), has been distributed in Burkina Faso since 2016 and is administered within the first year of age (at birth, 8 weeks, 12 weeks, and 16 weeks). Here, PV1-associated sero-reactivity was considered a biomarker for poliovirus vaccine uptake. The cumulative antibody response did not vary across age, sex, or time point (Figure 4; Supplemental Table 4). The most antigenic regions of poliovirus were localized in the VP1 region of the nuclear capsid protein (Figure 4). At the VP1 level, no notable difference in antibody response as a function of age, sex, or time points (Supplemental Table 5) were detected. Finally, biannual azithromycin treatments in preschool children did not lead to a change in PV1-associated antibody response compared with those treated with placebo (β coefficient: −53.51; 95% CI: −115.52 to 8.50; *P_adj_* = 0.34; Figure 4; Supplemental Table 4), regardless of sero-reactivity to SARS-CoV-2 S protein (Supplemental Table 4).

## DISCUSSION

As expected, the antibody profiles of preschool-aged children in Burkina Faso in 2023 were enriched for SARS-CoV-2-associated antibodies. Sero-reactivity to RSV appeared minimally changed between 2019 and 2023. The study also showed no detectable changes in the antibody profiles to PV1 over time.

Although sero-reactivity to SARS-CoV-2 was not associated with sex or community exposure to azithromycin mass distribution, it was negatively correlated with age, with the most robust sero-reactivity observed in children aged 12–23 months. This finding is consistent with a report that showed decreasing anti-spike antibody levels in older children.^14^ In particular, previously disease- and vaccine-naïve Taiwanese children aged 6 months to 2 years had higher anti-spike antibodies than children aged 2–5 years.^14^ The underlying cause was unclear, although the investigators hypothesized that the routine early childhood vaccination schedule for other viruses (e.g, measles, mumps, and polioviruses) results in heterogeneous and nonspecific sero-reactivity to SARS-CoV-2. In Burkina Faso, exclusive breastfeeding in infants decline after 6 months of age, when complementary food is introduced. Therefore, maternal antibodies from breast milk are unlikely to be a major contributor to the SARS-CoV-2 sero-reactivity in children aged 12–23 months.^15^

RSV-associated respiratory diseases are leading causes of childhood morbidity and mortality in sub-Saharan African countries.^16^ Before the emergence of SARS-CoV-2, the prevalence of RSV infections in Western African countries, including Burkina Faso, ranged from 16 to 32%.^17^ Current literature indicates changes in epidemiological trends in RSV infections. Specifically, RSV infections declined worldwide shortly after the declaration of the COVID-19 pandemic and atypically resurged during off-season periods as restrictions were lifted.^18^^–^^22^ Multiple potential mechanisms have been proposed to explain the observed epidemiological trends, including, but not limited to, reduced immune stimulation from lower viral exposures from school closures and various social distancing measures, SARS-CoV-2–related immune dysregulation, and immune interactions between SARS-CoV-2 and RSV infections.^23^ Regardless of driving etiology, our finding did not detect differences in RSV sero-reactivity in DBS collected from preschool children in 2022–2023 compared with those collected in 2019, even after adjusting for SARS-CoV-2 sero-reactivity. These results suggest that population-level immunity to RSV has not declined in this population of children from the Nouna region of Burkina Faso.

Sero-reactivity to PV1 was notable in the majority of children tested. Because healthcare is free for children under 5 years of age in Burkina Faso, vaccine uptake is generally high. According to World Health Organization immunization data, the official coverage for bOPV in Burkina Faso between 2018 and 2022 ranged from 91 to 96%.^24^ By 2023, Burkina Faso political instability led to highly variable vaccine coverage at the Centres de Santé et de Promotion Sociale level. However, given that the DBS samples were collected over a 2-month period beginning in December 2022 and included only children older than 12 months, PV1 sero-reactivity at the 36-month time point likely reflects the high national coverage documented for 2022. Consistent with reported vaccine coverage data from 2018 and 2022, no significant difference in PV1 sero-reactivity was observed between samples collected in 2019 and those collected in 2022.

The CHAT trial, a cluster-randomized controlled trial, randomized communities to receive either azithromycin or placebo distributed to children aged 1–59 months. This design provided a convenient platform to determine whether antibiotic administration modifies gut health and, in turn, improves immunogenicity of polio vaccine uptake in preschool children in Burkina Faso.^25^ In this study, no statistically significant difference in PV1 sero-reactivity was detected with mass azithromycin distributions. These findings are similar to results from a prior randomized controlled trial, in which infants in India received a 3-day course of azithromycin or placebo before receiving the poliovirus vaccine.^25^ The authors found that the sero-conversion rates were similar in the two treatment arms.^25^ Thus, in the Burkina Faso population, azithromycin mass drug distribution does not appear to have a large immunogenicity effect on sero-reactivity to PV1 or other viruses, including SARS-CoV-2 and RSV.

### Limitations.

The cross-sectional aspect of this population-based study limits the ability to evaluate the longitudinal changes in sero-reactivity within individual children to characterize waning immunity or increasing affinity to viral targets. Additionally, the association between bOPV administration and PV1 sero-reactivity could not be evaluated, as individual vaccination data were not collected in the CHAT study. Although the phage-displayed virome approach enables simultaneous characterization of sero-reactivity profiles of multiple viral targets in DBS, further work is needed to define immune correlates of protection. Incorporation of conventional serologic methods, such as ELISA and Multiplex Bead Assay, would further improve our understanding of sero-prevalence in this population. Finally, this exploratory study used a convenience set of samples, and as such, was not likely to detect statistically significant comparisons unless the effect sizes were large.

## CONCLUSION

This exploratory, cross-sectional, population-based study showed that the sero-reactivity to SARS-CoV-2 in preschool children in Burkinabè was age dependent. Mass drug administration did not appear to influence the sero-reactivity to viruses in these children. In addition, no large detectable effects in antibody profiles to RSV and PV1 were detected 3 years after the emergence of SARS-CoV-2. Future studies would benefit from larger sample sizes and a longitudinal design to improve the mechanisms underlying serological responses to viral infections in children in sub-Saharan Africa. PhIP-Seq using DBS has the potential to improve widespread monitoring of viral infections and serosurveillance at the regional level and within countries.

## Supplemental Materials

10.4269/ajtmh.25-0408 Supplemental Materials
