# Supplementary material for: Population-Based Viral Antibody Profiles of Preschool Children in Burkina Faso
Source: Am J Trop Med Hyg. 2025 Nov 6;114(1):145–52. doi: 10.4269/ajtmh.25-0408 (PMC12781471; doi:10.4269/ajtmh.25-0408)
Supplement: Supplemental Materials [file tpmd250408.SD1.pdf]

Supplementary Materials

Table of Contents

Supplementary Table 1. Spike Protein, All Model Estimates. *2*

Supplementary Table 2. SARS-CoV-2 Proteins, Model Estimate for Age, adjusted by Sex. *3*

Supplementary Table 3. RSV Proteome, All Model Estimates. *4*

Supplementary Table 4. Poliovirus 1, All Model Estimates. *5*

Supplementary Table 5. VP1, All Model Estimates. *6*

Supplementary Figure 1: Laboratory Workflow. *7*

Supplementary Table 1. Spike Protein, All Model Estimates.

Variable	Model Estimate (95% CI)	Unadjusted *P*-Value	FDR-Adjusted *P*-Value
Sex	4.63 (-11.90, 21.17)	0.579	0.579
Age, Adjusted by Sex	-1.43 (-2.03, -0.84)	<0.001	<0.001
Arms	-9.43 (-27.56, 8.71)	0.300	0.450

Abbreviations: CI = confidence interval, FDR = Benjamini-Hochberg False Discovery Rate. Model estimates and unadjusted p-values were generated using a linear mixed effects model, with cluster as the random effect. Each row represents a different model.

Supplementary Table 2. SARS-CoV-2 Proteins, Model Estimate for Age, adjusted by Sex.

Protein	Model Estimate (95% CI)	Unadjusted *P*-Value	FDR-Adjusted *P*-Value
ORF1ab	0.01 (-0.77, 0.79)	0.986	0.986
S	-1.43 (-2.03, -0.84)	<0.001	<0.001
ORF3a	0.02 (-0.23, 0.28)	0.853	0.938
E	0.00 (-0.00, 0.01)	0.328	0.726
M	-0.09 (-0.21, 0.03)	0.140	0.726
ORF6	-0.00 (-0.01, 0.01)	0.722	0.887
ORF7a	0.02 (-0.01, 0.05)	0.271	0.726
ORF7b	0.00 (-0.01, 0.01)	0.726	0.887
ORF8	-0.01 (-0.05, 0.02)	0.526	0.827
N	0.13 (-0.26, 0.51)	0.523	0.827
ORF9c	0.01 (-0.01, 0.02)	0.330	0.726

Abbreviations: CI = confidence interval, FDR = Benjamini-Hochberg False Discovery Rate. Model estimates and unadjusted p-values were generated using a linear mixed effects model, with cluster as the random effect. Each row represents a different model, with a different protein as the outcome.

Supplementary Table 3. RSV Proteome, All Model Estimates.

Variable	Model Estimate (95% CI)	Unadjusted *P*-Value	FDR-Adjusted *P*-Value
Sex (Baseline)	-10.67 (-47.65, 26.31)	0.567	0.680
Sex (36 Months)	43.15 (-25.23, 111.53)	0.213	0.426
Age (Baseline)	-0.20 (-1.77, 1.38)	0.805	0.805
Age (36 Months)	-1.00 (-3.67, 1.67)	0.457	0.680
Arm, Adjusted by Spike (36 Months)	54.55 (-19.12, 128.22)	0.142	0.426
Time Point	39.26 (-0.20, 78.72)	0.051	0.306

Abbreviations: CI = confidence interval, FDR = Benjamini-Hochberg False Discovery Rate. Model estimates and unadjusted p-values were generated using a linear mixed effects model, with cluster as the random effect. Each row represents a different model.

Supplementary Table 4. Poliovirus 1, All Model Estimates.

Variable	Model Estimate (95% CI)	Unadjusted *P*-Value	FDR-Adjusted *P*-Value
Sex (Baseline)	-49.90 (-112.12, 12.32)	0.114	0.342
Sex (36 Months)	14.43 (-47.23, 76.10)	0.643	0.821
Age (Baseline)	-0.52 (-3.06, 2.02)	0.684	0.821
Age (36 Months)	0.26 (-2.13, 2.64)	0.832	0.832
Arm, Adjusted by Spike (36 Months)	-53.51 (-115.52, 8.50)	0.089	0.342
Time Point	17.86 (-25.35, 61.07)	0.416	0.821

Abbreviations: CI = confidence interval, FDR = Benjamini-Hochberg False Discovery Rate. Model estimates and unadjusted p-values were generated using a linear mixed effects model, with cluster as the random effect. Each row represents a different model.

Supplementary Table 5. VP1, All Model Estimates.

Variable	Model Estimate (95% CI)	Unadjusted *P*-Value	FDR-Adjusted *P*-Value
Sex (Baseline)	30.37 (6.00, 54.73)	0.015	0.090
Sex (36 Months)	11.07 (-11.36, 33.50)	0.329	0.658
Age (Baseline)	0.26 (-0.76, 1.28)	0.614	0.915
Age (36 Months)	0.05 (-0.83, 0.93)	0.910	0.915
Arm, Adjusted by Spike (36 Months)	-17.54 (-42.81, 7.73)	0.168	0.504
Time Point	0.89 (-15.61, 17.40)	0.915	0.915

Abbreviations: CI = confidence interval, FDR = Benjamini-Hochberg False Discovery Rate. Model estimates and unadjusted p-values were generated using a linear mixed effects model, with cluster as the random effect. Each row represents a different model.

Supplementary Figure 1: Laboratory Workflow.

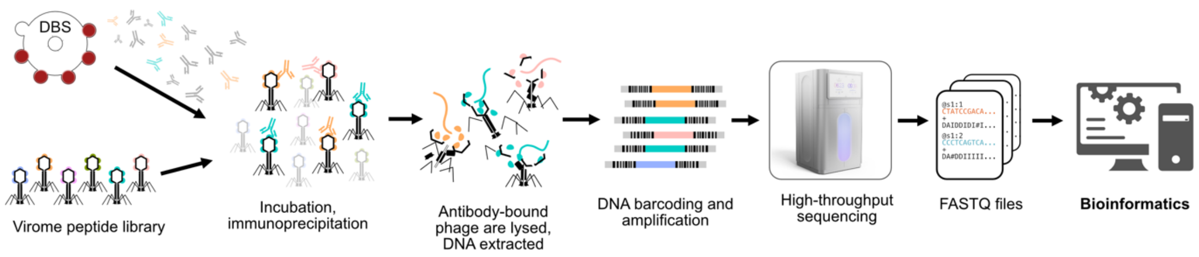

Antibodies from dried blood spots (DBS) were incubated with the laboratory-generated phage-displayed viral peptidome. Phage-bound antibodies were immunoprecipitated and subjected to high-throughput sequencing and subsequent bioinformatics.
